# Supplementary material for: Causal Relationship between Aging and Anorexia Nervosa: A White-Matter-Microstructure-Mediated Mendelian Randomization Analysis
Source: Biomedicines. 2024 Aug 16;12(8):1874. doi: 10.3390/biomedicines12081874 (PMC11351342; doi:10.3390/biomedicines12081874)
Supplement: Supplementary file 1 [file biomedicines-12-01874-s001.zip › Supplementary Figure/Figure S4/Singe SNP plot of MR analysis for TL to ubm-b-1698.pdf]

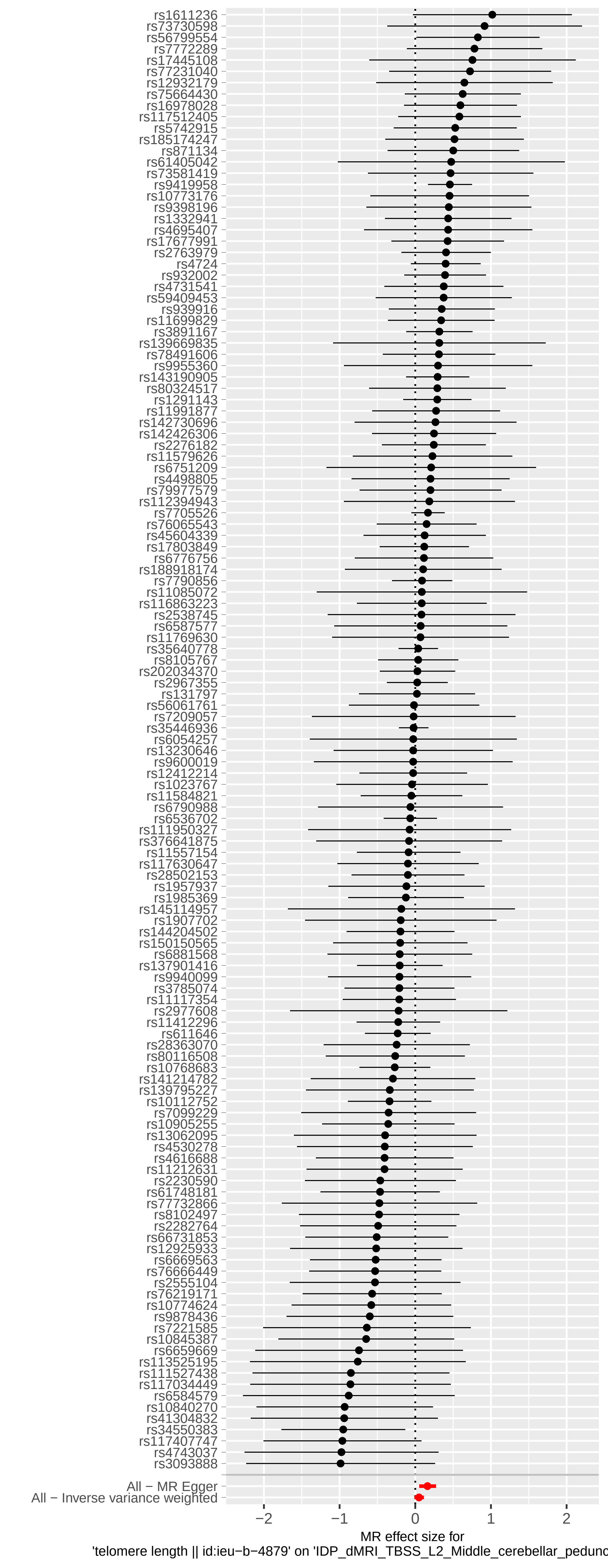

rs1611236
rs73730598
rs56799554
rs7772289
rs17445108
rs77231040
rs12932179
rs75664430
rs16978028
rs117512405
rs5742915
rs185174247
rs871134
rs61405042
rs73581419
rs9419958
rs10773176
rs9398196
rs1332941
rs4695407
rs17677991
rs2763979
rs4724
rs932002
rs4731541
rs59409453
rs939916
rs11699829
rs3891167
rs139669835
rs78491606
rs9955360
rs143190905
rs80324517
rs1291143
rs11991877
rs142730696
rs142426306
rs2276182
rs11579626
rs6751209
rs4498805
rs79977579
rs112394943
rs7705526
rs76065543
rs45604339
rs17803849
rs6776756
rs188918174
rs7790856
rs11085072
rs116863223
rs2538745
rs6587577
rs11769630
rs35640778
rs8105767
rs202034370
rs2967355
rs131797
rs56061761
rs7209057
rs35446936
rs6054257
rs13230646
rs9600019
rs12412214
rs1023767
rs11584821
rs6790988
rs6536702
rs111950327
rs376641875
rs11557154
rs117630647
rs28502153
rs1957937
rs1985369
rs145114957
rs1907702
rs144204502
rs150150565
rs6881568
rs137901416
rs9940099
rs3785074
rs11117354
rs2977608
rs11412296
rs611646
rs28363070
rs80116508
rs10768683
rs141214782
rs139795227
rs10112752
rs7099229
rs10905255
rs13062095
rs4530278
rs4616688
rs11212631
rs2230590
rs61748181
rs77732866
rs8102497
rs2282764
rs66731853
rs12925933
rs6669563
rs76666449
rs2555104
rs76219171
rs10774624
rs9878436
rs7221585
rs10845387
rs6659669
rs113525195
rs111527438
rs117034449
rs6584579
rs10840270
rs41304832
rs34550383
rs117407747
rs4743037
rs3093888
All − MR Egger
All − Inverse variance weighted
−2
−1
0
1
2
MR effect size for
'telomere length || id:ieu−b−4879' on 'IDP_dMRI_TBSS_L2_Middle_cerebellar_peduncl
